# Human Umbilical Cord Mesenchymal Stem Cells Attenuate Ocular Hypertension-Induced Retinal Neuroinflammation via Toll-Like Receptor 4 Pathway

**DOI:** 10.1155/2019/9274585

**Published:** 2019-10-15

**Authors:** Shangli Ji, Jie Xiao, Jian Liu, Shibo Tang

**Affiliations:** ^1^Aier School of Ophthalmology, Central South University, Changsha, Hunan, China; ^2^Aier Eye Institute, Changsha, Hunan, China; ^3^Department of Anatomy and Neurobiology, Xiangya School of Medicine, Central South University, Changsha, Hunan, China

## Abstract

Glaucoma is characterized by progressive, irreversible damage to the retinal ganglion cells (RGCs) and their axons. Our previous study has shown that the intravitreal transplantation of human umbilical cord mesenchymal stem cells (hUC-MSCs) reveals a neuroprotective role in microsphere injection-induced ocular hypertension (OHT) rat models. The protection is related to the modulation of glial cells, but the mechanisms are still unknown. The purpose of the present study is to clarify the potential neuroinflammatory mechanisms involved in the neuroprotective role of hUC-MSCs. OHT models were established with SD rats through intracameral injection of polystyrene microbeads. The animals were randomly divided into three groups: the normal group, the OHT+phosphate-buffered saline (PBS) group, and the OHT+hUC-MSC group. Retinal morphology was evaluated by measuring the inner retinal thickness via optical coherence tomography (OCT). Retinal cell apoptosis was examined by TUNEL staining and Bax expression 14 days following hUC-MSC transplantation. The expression levels of glial fibrillary acidic protein (GFAP), ionized calcium binding adapter molecule 1 (iba-1), and toll-like receptor 4 (TLR4) were assessed via immunohistochemistry, real-time quantitative PCR, and Western blot. RNA and proteins were extracted 14 days following transplantation, and the expression levels of the TLR4 signaling pathways and proinflammatory cytokines—myeloid differentiation factor 88 (MyD88), IL-1*β*, IL-6, and TNF-*α*—were determined. OCT showed that the intravitreal transplantation of hUC-MSCs significantly increased the inner thickness of the retina. A TUNEL assay and the expression of Bax suggested that the apoptosis of retinal cells was decreased by hUC-MSCs 14 days following transplantation. Intravitreal hUC-MSC transplantation resulted in a decreased expression of GFAP, iba-1, TLR4, MyD88, IL-1*β*, IL-6, and TNF-*α* 14 days following transplantation. In addition, via in vitro experiments, we found that the increased expression of the TLR4 signaling pathway induced by lipopolysaccharide (LPS) was markedly decreased after hUC-MSCs were cocultured with rMC-1 and BV2 cells. These findings indicate that hUC-MSC transplantation attenuates OHT-induced retinal neuroinflammation via the TLR4 pathway.

## 1. Introduction

Glaucoma is characterized by progressive, irreversible damage to the retinal ganglion cells (RGCs) and their axons [1]. Glaucoma is an age-related multifactorial neurodegenerative disease, the most common risk factor of which is elevated intraocular pressure (IOP). However, 15-25% of patients continue to lose vision despite appropriate IOP control. As a consequence, there may be other mechanisms involved in the progression of the disease [2]. Recently, an increasing number of literatures have suggested that neuroinflammation is a vital process in glaucoma [3–6]. Neuroinflammation is generated by the resident innate immune cells, such as astrocytes and Müller cells, as well as microglia in the retina and optic nerve [4, 7]. Glial cells are recognized as playing vital immunological roles within the retina, and their participation in glaucoma progression has been reported [8, 9]. Formichella et al. found that astrocyte reactivity occurs both in glaucomatous retina and retina with glaucoma risk factors [10]. At the same time, microglial proliferation and activation have been found in glaucoma in both animal models and human beings [11, 12]. An increasing amount of animal studies have demonstrated that the modulation of neuroinflammation might be a promising therapeutic strategy. Retinal and optic nerve neurodegeneration in a glaucoma mouse model was demonstrated to be related to microglial activation [12]. Madeira et al. found that caffeine could help in preventing the retinal microglia-related neuroinflammatory response and attenuating the RGC loss in OHT animals [13]. In addition, they also demonstrated that A2AR antagonists might be a potential therapeutic choice to regulate glaucomatous disorders by controlling microglia-mediated retinal neuroinflammation [2].

Toll-like receptor 4 (TLR4) is reported to be upregulated in human glaucoma, and the glaucomatous retina stress possibly initiates the immunostimulatory pathway via glial TLR4 [14]. Previous studies have indicated that in some populations, the increased risk of glaucoma is associated with certain alleles of the TLR4 gene [15, 16]. Tenascin C, one of the endogenous ligands for TLR4, is highly upregulated in glaucomatous eyes, both in human beings and animal models [17, 18]. These abovementioned findings indicate that glial cells and TLR4-related signaling pathways might be active participants in the process of glaucoma. Previous studies have suggested that mesenchymal stem cells (MSCs) are capable of regulating the function of microglia and astrocytes, both directly through MSCs and indirectly via MSC-derived exosomes (MSC-exo) [19–22]. MSCs and MSC-exo regulate the polarization of microglia and exert an antiproliferative effect on lipopolysaccharide- (LPS-) stimulated microglia [19, 20]. MSCs and MSC-exo reduce neurotoxic A1 astrocytes and exert anti-inflammatory and neuroprotective effects [21, 22]. Human umbilical cord mesenchymal stem cells (hUC-MSCs) have been extensively studied as a promising therapy for eye diseases [23–27] and have also been shown to have immunomodulation properties [23, 24]. A study has disclosed that hUC-MSCs exhibit anti-inflammatory effects by inhibiting TLR4 signaling pathway activation in renal fibrosis [28]. It has also been reported that hUC-MSC-exosomes reduce burn-induced inflammation by downregulating the TLR4 signaling pathway [29]. Recently, with the increase in reports on the immunomodulatory prospects of hUC-MSCs, we and others have explored and demonstrated the potential of hUC-MSCs to modulate the reactivity of glial cells in glaucoma [30]. However, the mechanisms underlying the function remain unclear. In the present study, we therefore sought to test whether hUC-MSCs attenuate retinal neuroinflammation via the downregulation of the TLR4 signaling pathway.

## 2. Methods

### 2.1. Animals and In Vivo Experimental Design

**Figure 1:**
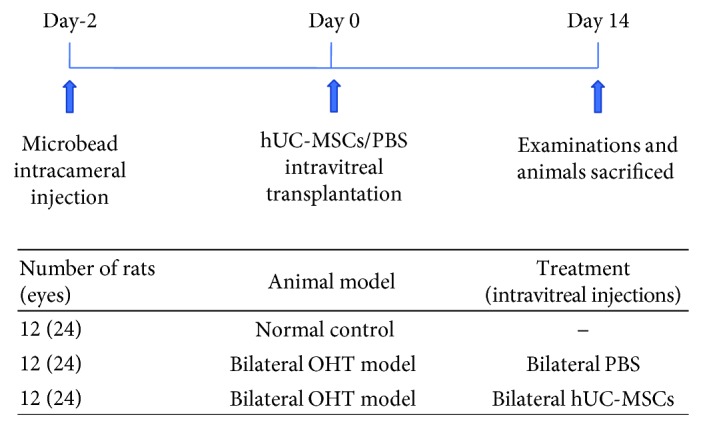
Experimental design. Time schedule of the experimental design detailing the times when the OHT model, intravitreal treatments, and tissue collections were performed.

All procedures and postsurgical care were in accordance with the ARVO Statement for the Use of Animals in Ophthalmic and Vision Research and were approved by the Committee on the Use of Animals in the Central South University. All animals used in our present experiments were twelve-week-old (200-250 g) male Sprague-Dawley (SD) rats. The in vivo experimental design is detailed in Figure 1. Eighteen rats (36 eyes) were randomly divided into three groups: the normal group (12 eyes), the OHT+PBS group (12 eyes), and the OHT+hUC-MSC group (12 eyes). Both eyes in each animal received the same treatment. Animals were anesthetized with pentobarbital sodium (50 mg/kg) via intraperitoneal injections (Yitai, China).

### 2.2. hUC-MSCs, OHT Model, and Intravitreal Cell Transplantation

Cryopreserved hUC-MSCs were rapidly thawed in a 37°C water bath immediately prior to employment. As described in our previous study, hUC-MSCs were cultured in the full medium containing Dulbecco's modified Eagle's medium (DMEM) (Gibco, USA), 10% fetal bovine serum (FBS) (Gibco, USA), 1% penicillin/streptomycin (Gibco, USA), and 1% 2 mM L-glutamine (Gibco, USA) [30]. Cells were cultured in a humidified atmosphere of 5% CO_2_ and 95% air at 37°C, and passages 3-5 were used in the experiments. The establishment of the OHT model and hUC-MSC transplantation were performed as described in our previous study [30]. Based on our previous study, elevated IOP was established via bilateral anterior chamber injection of a 10 *μ*L microsphere (10 *μ*m diameter, Thermo Fisher Scientific, USA) suspension with a concentration of 1 × 10^4^ microspheres/*μ*L. Two days later, a 5 *μ*L 10^5^ hUC-MSC suspension in PBS was bilaterally transplanted to the vitreous cavity of the OHT rats. As the control, 5 *μ*L sterile PBS was used as equivalent in the OHT rat eyes. IOP was measured every four days in both eyes with a tonometer (TonoLab, Finland) as previously described [30]. IOP measurements were obtained for 2 weeks to confirm OHT in rats for 2 weeks (Supplementary Materials: [Supplementary-material supplementary-material-1]).

### 2.3. Measurement of Inner Retinal Thickness

Optical coherence tomography (OCT) was employed to measure the rat inner retinal thickness, including the retinal nerve fiber layer/retinal ganglion cell layer (RNFL/RGCL), inner plexiform layer (IPL), and inner nuclear layer (INL). Fundus imaging and OCT were performed on rats using a Micron IV (Phoenix Research Labs, USA) with a contact lens specifically designed for rat OCT. Fundus and OCT pictures of retina around the optic nerve head were taken, and the Insight software (Phoenix Research Labs, USA) was used to quantify the inner retinal thickness. Three different positions of every OCT image were respectively measured, and the three chosen positions (at the 648, 1728, and 2808 dots of the horizontal coordinate) were the same within all of the OCT images. The three values for each eye were then averaged to obtain the inner retinal thickness.

### 2.4. rMC-1 Cell Culture and Stimulation

Rat Müller cells (rMC-1) were purchased from BeNa culture collection (BeNa, China). rMC-1 cells were cultured in DMEM containing 10% FBS and 1% penicillin-streptomycin at 37°C in a humidified incubator containing 5% CO_2_. rMC-1 cells were cultured in FBS-free medium for 24 h after reaching 60% confluency and then stimulated with 1 *μ*g/mL lipopolysaccharides (LPS) (Sigma-Aldrich, USA).

### 2.5. BV2 Microglia Cell Culture and Stimulation

A murine microglial cell line (BV2) was purchased from the National Infrastructure of Cell Line Resource (China). BV2 cells were cultured in DMEM containing 10% FBS and 1% penicillin-streptomycin at 37°C in a humidified incubator containing 5% CO_2_. BV2 cells were cultured in FBS-free medium for 24 h after reaching 60% confluency and then stimulated with 1 *μ*g/mL lipopolysaccharide LPS.

### 2.6. hUC-MSC/rMC-1 Cocultures and hUC-MSC/BV2 Cocultures

rMC-1 and BV2 cells were independently stimulated with 1 *μ*g/mL LPS with or without hUC-MSCs using a transwell coculture system (Corning, USA) independently in a humidified incubator at 37°C for 24 h. Cell culture inserts with a 0.4 *μ*m polyester membrane were used.

### 2.7. TUNEL Staining

The eyes were enucleated and fixed with 4% paraformaldehyde (Sinopharm, China) overnight at 4°C and were then dehydrated with 15% and 30% sucrose followed by being embedded in an optimum cutting temperature compound (Sakura, USA). Eye sections (8 *μ*m thick) were cut vertically to the cornea. In accordance with the manufacturer's instructions, the DeadEnd Colorimetric TUNEL System (Promega, USA) was used to assay the apoptosis of the retinal tissue. Ten sections of each eye sample were included in the count of the TUNEL staining assay.

### 2.8. Real-Time Quantitative Reverse Transcription Polymerase Chain Reaction (RT-qPCR)

Total RNA was isolated from retinas using the TRIzol Reagent and then reverse transcribed with the All-in-One First-Strand cDNA Synthesis Kit to synthesize cDNA according to the instructions provided by the manufacturer. Real-time PCR was performed in the LightCycler 96 (Roche, Germany) using the SYBR GREEN PCR MasterMix. Relative gene expression was analyzed by comparison with the gene *β*-actin (*Actb*) (all reagents were obtained from Invitrogen, USA). The primers are listed in Table 1.

### 2.9. Western Blot Analysis

After sacrifice, the eyeballs were enucleated and immersed in PBS. For retina isolation, the anterior segments were removed by incising the cornea, and the retinas were then isolated in PBS. The retinas were collected and lysed in ice-cold RIPA lysis buffer (Solarbio Life Sciences, China). Next, 30 *μ*g protein in loading buffer was heated for 8 min at 99°C, followed by loading onto the 12% polyacrylamide gels. Protein was then transferred onto a nitrocellulose (NC) membrane, followed by incubation in 5% fat-free milk blocking buffer for 1 h on a shaking table at room temperature. It was then incubated overnight at 4°C with the primary antibody. Primary antibodies were directed against Bax (1 : 500), GAPDH (1 : 1000), GFAP (1 : 500), iba-1 (1 : 1000), TLR4 (1 : 500), MyD88 (1 : 500), IL-1*β* (1 : 1000), IL-6 (1 : 1000), TNF-*α* (1 : 1000), and actin (1 : 1000). The NC membrane was then diluted in the secondary antibody (Dylight 800, 1 : 5000) and incubated for 1 h at room temperature. Bands were analyzed with an Odyssey Fc Imaging System (LI-COR Biosciences, USA).

### 2.10. Immunohistochemistry Staining

The eye sections (8 *μ*m thick) were prepared with the same method described above. Eye sections were treated with 0.1% Triton X-100 for 30 min at room temperature before blocking in 10% goat serum (Beyotime, China) for 1 h (room temperature). Eye sections were then incubated overnight at 4°C with the primary antibody. Primary antibodies were directed against GFAP (1 : 200), iba-1 (1 : 100), TLR4 (1 : 100), and CD16 (1 : 100). Alexa Fluor-conjugated secondary antibodies (Jackson ImmunoResearch Inc., USA) and biotin-conjugated secondary antibody (Zhongshanjinqiao, China) were used next. After washing with PBS, eye sections were stained with DAPI (Vector, USA) or DAB (Zhongshanjinqiao, China). Images of the eye sections were captured on a digital microscope (Olympus, Japan). For the IHC quantification, images were analyzed with the IHC Profiler Plugin of ImageJ Software, and highly positive and positive scores were used for the calculation of each eye sample [31].

### 2.11. Data Analysis and Statistics

GraphPad software (La Jolla, USA) was used to analyze data, and data are presented as mean ± SEM. The differences between groups were analyzed by one-way ANOVA followed by the Tukey test for multiple comparisons. A *P* value less than 0.05 was considered statistically significant.

## 3. Results

### 3.1. hUC-MSC Transplantation Preserves the Inner Retinal Thickness

**Figure 2:**
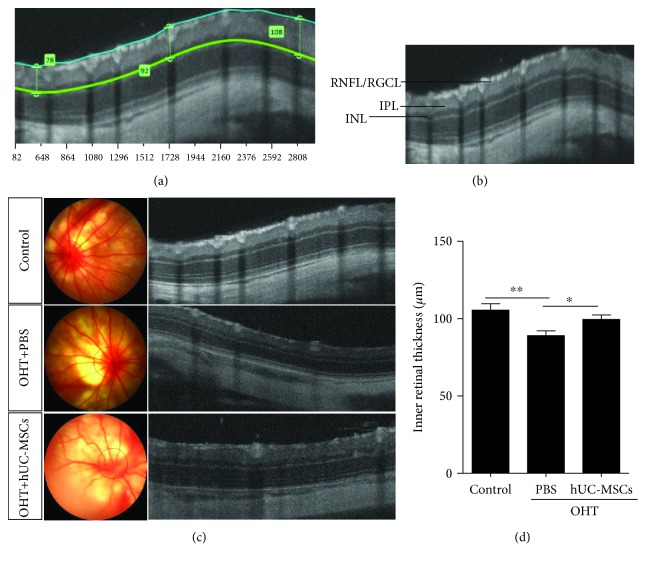
hUC-MSC transplantation increases the inner retinal thickness in OHT-induced rats. (a) Method of measurement of the inner retina thickness via OCT images. (b) RNFL/RGCL (retinal nerve fiber layer/retinal ganglion cell layer); IPL (inner plexiform layer); INL (inner nuclear layer). (c) Two weeks after hUC-MSC injection. Representative fundus photography and OCT images of normal eyes, OHT+PBS eyes, and OHT+hUC-MSC eyes. (d) Results of inner retinal thickness quantification (*n* = 4/group; ^∗^*P* < 0.05 and ^∗∗^*P* < 0.01 compared with one-way ANOVA with Turkey's post hoc tests).

To investigate the effects of hUC-MSCs on retinal damage, the inner retinal thickness around the optic nerve head was examined by using the OCT two weeks after intravitreal injection of hUC-MSCs and PBS in the OHT rats. Similar to our previous study, which reported that transplanted hUC-MSCs had preserved RGC function [30], morphology analysis revealed that hUC-MSC transplantation increases the inner retinal thickness (Figures 2(a)–2(c)). In OHT animals, the inner retinal thickness (89.09 ± 3.04 *μ*m) was significantly decreased (*P* = 0.0058) compared with that of the normal group (109.6 ± 3.87 *μ*m). Compared with the PBS-treated OHT eyes, the inner retinal thickness in the hUC-MSC-treated OHT rats (105.4 ± 3.37 *μ*m) significantly increased (*P* = 0.0187) (Figure 2(d)). These abovementioned results indicate that transplanted hUC-MSCs in OHT rats exhibit a neuroprotective effect.

### 3.2. Engrafted hUC-MSCs Attenuate Apoptosis in the Retinas

**Figure 3:**
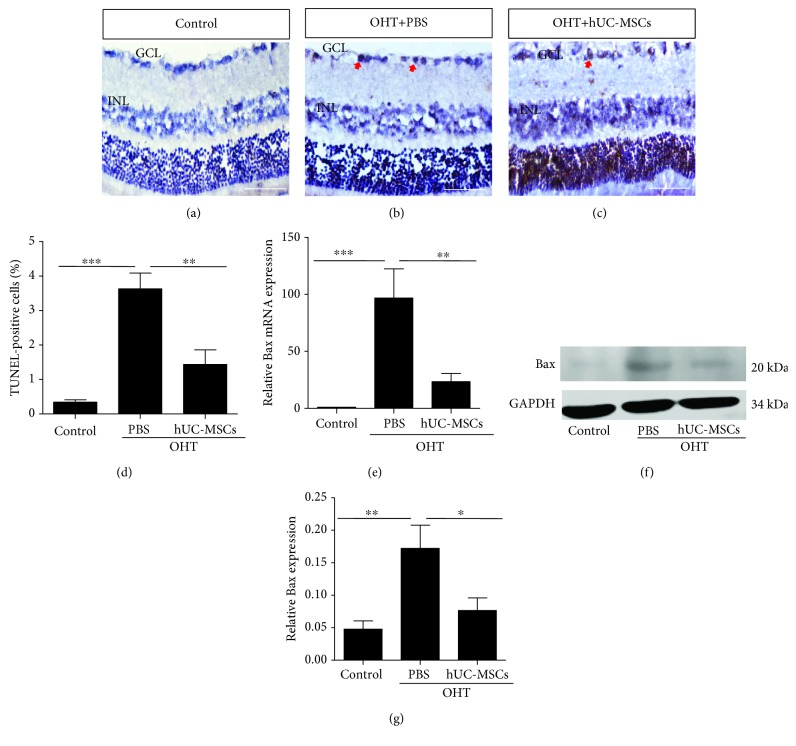
hUC-MSC transplantation decreases the apoptosis of retinal cells in OHT-induced rats. Two weeks after hUC-MSC injection. (a–c) Representative images of TUNEL staining in retinas from normal eyes, OHT+PBS eyes, and OHT+hUC-MSC eyes. The positive cells in the GCL and ILN of retinas were brown, and the arrow indicates positive TUNEL staining. (d) Quantification of TUNEL-positive cells (*n* = 4/group; ^∗∗^*P* < 0.01 and ^∗∗∗^*P* < 0.001 compared with one-way ANOVA with Turkey's post hoc tests). (e) Relative Bax gene expression that was normalized to GAPDH in retinas from normal eyes, OHT+PBS eyes, and OHT+hUC-MSC eyes (*n* = 4/group; ^∗∗^*P* < 0.01 and ^∗∗∗^*P* < 0.001 compared with one-way ANOVA with Turkey's post hoc tests). (f) Western blot analysis for Bax protein from normal eyes, OHT+PBS eyes, and OHT+hUC-MSC eyes. (g) Relative Bax protein expression that was normalized to GAPDH (*n* = 4/group; ^∗^*P* < 0.05 and ^∗∗^*P* < 0.01 compared with one-way ANOVA with Turkey's post hoc tests). Scale bar: 100 *μ*m.

To investigate whether engrafted hUC-MSCs affect the apoptosis of cells within the OHT eyes, TUNEL analysis was performed on retinal cryosections (Figures 3(a)–3(d)), and the expression of Bax was assayed in real-time PCR and western blot, respectively, two weeks after the transplantation of hUC-MSCs. Compared with the normal group (0.34 ± 0.07%), in OHT animals, the % TUNEL (3.63 ± 0.46%) was significantly increased (*P* < 0.0001). hUC-MSC-treated OHT retinas (1.44 ± 0.43%) had a significant (*P* = 0.0019) % TUNEL reduction compared with the PBS-treated OHT rats (Figure 3(g)). Similarly, Figures 3(e)–3(g) reveal that hUC-MSC-treated OHT retinas downregulated the Bax expression both in the gene (*P* = 0.0027, PBS-treated OHT vs. hUC-MSC-treated OHT) and protein (*P* = 0.0485, PBS-treated OHT vs. hUC-MSC-treated OHT) levels. These results demonstrate that transplanted hUC-MSCs can inhibit the apoptosis induced by OHT.

### 3.3. Engrafted hUC-MSCs Modulate Glial Reactivity and TLR4 Expression in the Retinas

**Figure 4:**
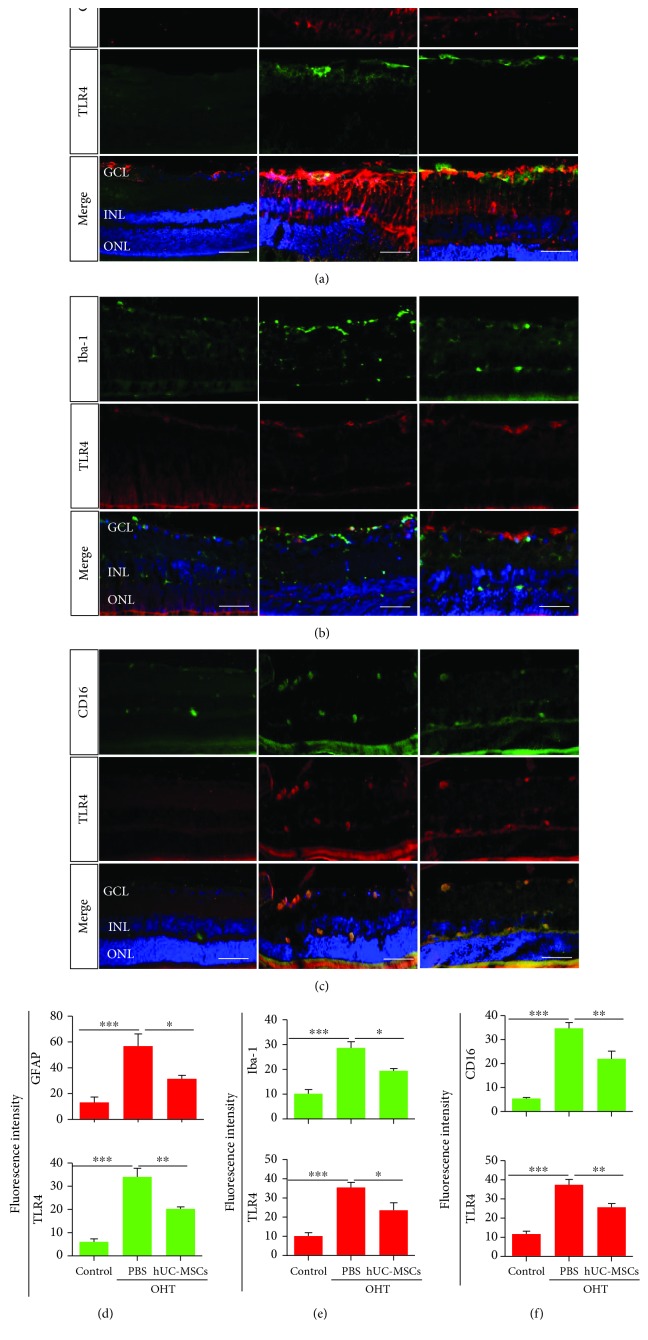
hUC-MSC transplantation modulates glial cell reactivity and TLR4 expression in the retinas of OHT-induced rats. Two weeks after hUC-MSC injection. (a) Double immunostaining of astrocytic marker GFAP (red) and TLR4 (green) in the control group, the PBS-treated OHT group, and the hUC-MSC-treated OHT group. Astrocyte activation was assessed by GFAP. TLR4 was colocalized in Müller cells and astrocytes (yellow). (b) Double immunostaining of microglial marker iba-1 (green) and TLR4 (red) in the control group, the PBS-treated OHT group, and the hUC-MSC-treated OHT group. TLR4 was poorly colocalized in iba-1-positive microglia. (c) Double immunostaining of active microglial marker CD16 (green) and TLR4 (red) in the control group, the PBS-treated OHT group, and the hUC-MSC-treated OHT group. TLR4 was colocalized in active microglia. (d) Fluorescence intensity of GFAP and TLR4 (*n* = 4/group; ^∗^*P* < 0.05, ^∗∗^*P* < 0.01, and ^∗∗∗^*P* < 0.001 compared with one-way ANOVA with Turkey's post hoc tests). (e) Fluorescence intensity of iba-1 and TLR4 (*n* = 4/group; ^∗^*P* < 0.05 and ^∗∗∗^*P* < 0.001 compared with one-way ANOVA with Turkey's post hoc tests). (f) Fluorescence intensity of GFAP and TLR4 (*n* = 4/group; ^∗∗^*P* < 0.01 and ^∗∗∗^*P* < 0.001 compared with one-way ANOVA with Turkey's post hoc tests). Scale bar: 50 *μ*m.

To examine whether engrafted hUC-MSCs affect glial activation and TLR4 expression in OHT eyes, the expression of GFAP, iba-1, CD16, and TLR4 was investigated via immunohistochemistry two weeks after hUC-MSC transplantation. In the retina, both astrocyte and microglial cells are quiescent in a normal situation, and they will become reactive once they are stimulated by various factors. Astrocytes present proliferation and exhibit thickened astrocytic processes, as well as increased GFAP-immunoreactivity [32]. When microglia become reactive, they exhibit morphological changes, including a rounded shape [33], increased somal size, and increased expression of surface markers such as CD16/32, CD86, and CD68 [34]. In our present study, compared with the control group, the expression of GFAP (*P* = 0.0007) and TLR4 (*P* < 0.0001) of the PBS-treated OHT group was increased, and TLR4 was colocalized in reactive astrocytes. However, hUC-MSC transplantation resulted in reduced GFAP (*P* = 0.0499) and TLR4 (*P* = 0.0016) expression compared with the PBS-treated OHT group (Figures 4(a) and 4(d)). Meanwhile, compared with the control group, the morphology of the iba-1-positive microglia of the PBS-treated OHT group was also altered, but the colocalization of iba-1 and TLR4 in the microglia was poor. hUC-MSC transplantation resulted in reduced iba-1 (*P* = 0.0310) and TLR4 (*P* = 0.0484) expression compared with the PBS-treated OHT group (Figures 4(b) and 4(e)). Next, we examined the expression of TLR4 in reactive microglia. Compared with the control group, the expression of CD16 (*P* < 0.0001) and TLR4 (*P* < 0.0001) of the PBS-treated OHT group was increased, and TLR4 was colocalized in reactive microglia. hUC-MSC transplantation resulted in reduced CD16 (*P* = 0.0041) and TLR4 (*P* = 0.0091) expression compared with the PBS-treated OHT group (Figures 4(c) and 4(f)). As illustrated in Figure 4, the reactive phenotype of astrocytes, microglial morphology, and TLR4 expression were altered after hUC-MSC treatment. This suggests that hUC-MSC transplantation modulates glial reactivity and TLR4 expression in OHT eyes.

### 3.4. Engrafted hUC-MSCs Decrease the Expression of TLR4, GFAP, and Iba-1

**Figure 5:**
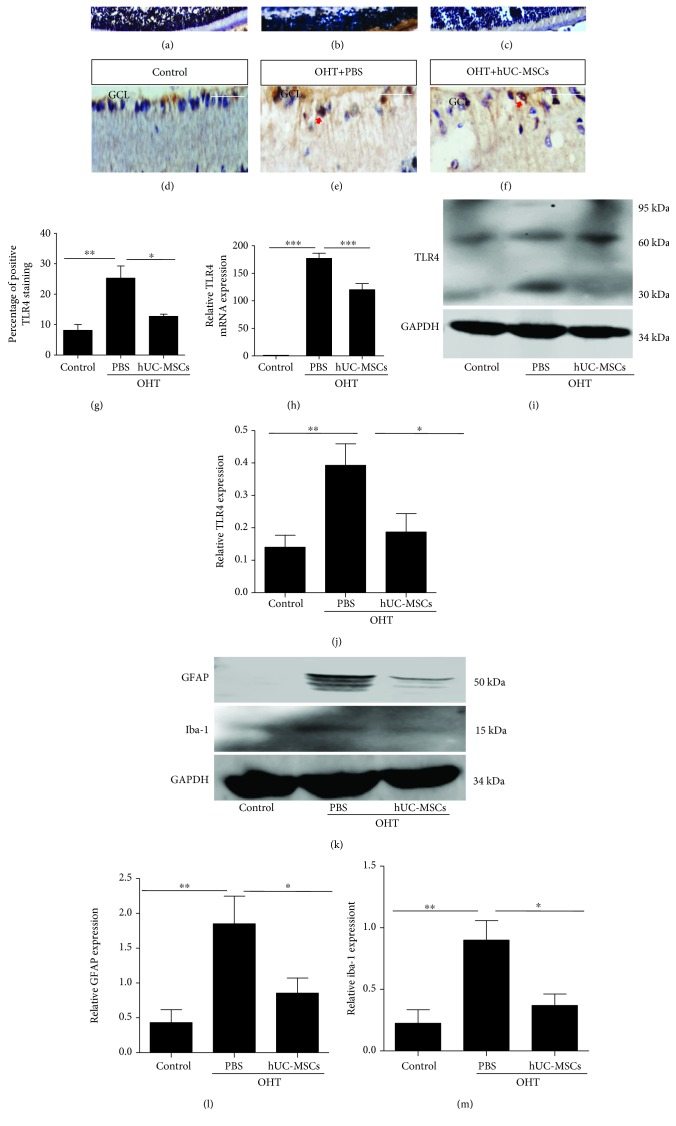
hUC-MSC transplantation decreases GFAP, iba-1, and TLR4 expression in the retinas of OHT-induced rats. Two weeks after hUC-MSC injection. (a–c) TLR4 immunohistochemistry staining from normal eyes, OHT+PBS eyes, and OHT+hUC-MSC eyes; (d–f) amplification of the corresponding groups. The arrow indicates positive TLR4 staining. (g) Quantification of TLR4 staining in retinas (*n* = 4/group; ^∗^*P* < 0.05 and ^∗∗^*P* < 0.01 compared with one-way ANOVA with Turkey's post hoc tests). (h) Relative TLR4 gene expression that was normalized to GAPDH in retinas from normal eyes, OHT+PBS eyes, and OHT+hUC-MSC eyes (*n* = 4/group; ^∗∗∗^*P* < 0.001 compared with one-way ANOVA with Turkey's post hoc tests). (i) Western blot analysis for TLR4 protein from normal eyes, OHT+PBS eyes, and OHT+hUC-MSC eyes. (j) Relative TLR4 protein expression that was normalized to GAPDH (*n* = 4/group; ^∗^*P* < 0.05 and ^∗∗^*P* < 0.01 compared with one-way ANOVA with Turkey's post hoc tests). (k) Western blot analysis of GFAP and iba-1 protein from normal eyes, OHT+PBS eyes, and OHT+hUC-MSC eyes. (l and m) Relative GFAP and iba-1 protein expression that was normalized to GAPDH (*n* = 4/group; ^∗^*P* < 0.05 and ^∗∗^*P* < 0.01 compared with one-way ANOVA with Turkey's post hoc tests). Scale bar: 100 *μ*m.

To examine whether engrafted hUC-MSCs affect TLR4 expression in OHT eyes, TLR4 expression was respectively assayed in immunohistochemistry, real-time PCR, and western blot two weeks after the transplantation of hUC-MSCs. In OHT animals, the TLR4 expression was significantly increased compared with the normal rats (*P* = 0.0056). Transplantation of hUC-MSCs in OHT eyes significantly reduced TLR4 (*P* = 0.0406) compared with the PBS-injected OHT eyes (Figures 5(a)–5(g)). Figures 5(h)–5(j) show that hUC-MSC-treated OHT retinas downregulated the TLR4 expression in both the gene (*P* = 0.0004) and protein levels (*P* = 0.0400). Figures 5(k)–5(m) suggest that the decreased protein expression of GFAP (*P* = 0.0500) and iba-1 (*P* = 0.0487) was found in the hUC-MSC-treated group. These results suggest that transplanted hUC-MSCs in OHT eyes decrease the expression of TLR4, GFAP, and iba-1.

### 3.5. Transplantation of hUC-MSCs Decreases OHT-Induced TLR4 Signaling Pathway Reactivity

**Figure 6:**
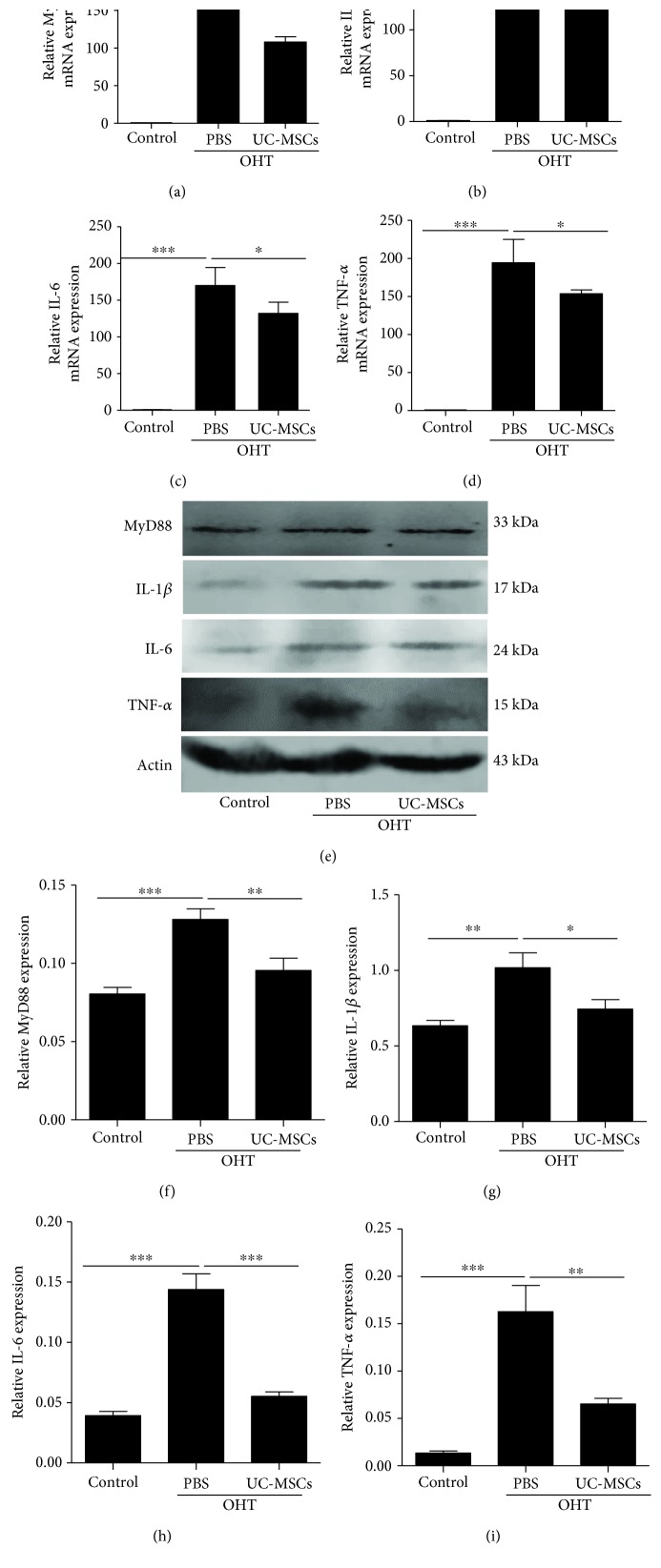
hUC-MSC transplantation decreases the expression of TLR4 pathway-related agents and proinflammatory mediators in the OHT-induced rats. Two weeks after hUC-MSC injection. (a–d) Relative gene expression of MyD88, IL-1*β*, IL-6, and TNF-*α* that was normalized to GAPDH in retinas from normal eyes, OHT+PBS eyes, and OHT+hUC-MSC eyes (*n* = 4/group; ^∗^*P* < 0.05 and ^∗∗∗^*P* < 0.001 compared with one-way ANOVA with Turkey's post hoc tests). (e) Western blot analysis for MyD88, IL-1*β*, IL-6, and TNF-*α* from normal eyes, OHT+PBS eyes, and OHT+hUC-MSC eyes. (f–i) Relative protein expression of MyD88, IL-1*β*, IL-6, and TNF-*α* that was standardized to actin (*n* = 4/group; ^∗^*P* < 0.05, ^∗∗^*P* < 0.01, and ^∗∗∗^*P* < 0.001 compared with one-way ANOVA with Turkey's post hoc tests).

The preceding results indicate that the expression and activation of glial cells, as well as TLR4 expression, are decreased after hUC-MSC transplantation. Next, we examined the downstream expression of the TLR4 signaling 14 days after hUC-MSC transplantation, which included MyD88 and proinflammatory mediators (IL-1*β*, IL-6, and TNF-*α*). As shown in Figure 6, the levels of MyD88 and proinflammatory cytokines were both determined at the gene and protein levels. Compared with the PBS-treated OHT eyes, hUC-MSC transplantation in OHT eyes significantly reduced the expression of MyD88 and proinflammatory mediators. These results suggest that transplanted hUC-MSCs in OHT eyes decrease MyD88 and proinflammatory cytokines.

### 3.6. hUC-MSCs Modulate TLR4 Pathway-Related Agents and Proinflammatory Mediator Levels in LPS-Treated rMC-1 Cells

**Figure 7:**
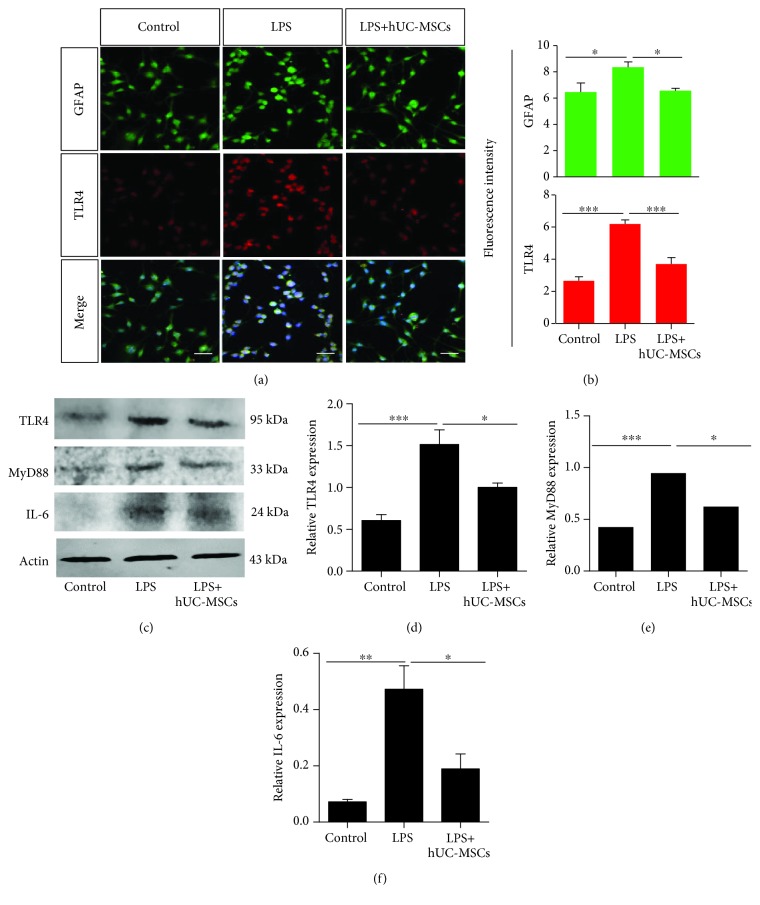
hUC-MSCs modulate TLR4 pathway-related agents and proinflammatory mediator levels in LPS-treated rMC-1 cells. (a and b) Double staining for GFAP (green) and TLR4 (red) showing colocalization of GFAP and TLR4. The fluorescence intensity showing reduced expression of GFAP and TLR4 in LPS-treated rMC-1 cells after hUC-MSC treatment (*n* = 4/group; ^∗^*P* < 0.05 and ^∗∗∗^*P* < 0.001 compared with one-way ANOVA with Turkey's post hoc tests). (c and d) Quantification of TLR4, MyD88, and IL-6 expression by western blot analysis showing a significant decrease in TLR4, MyD88, and IL-6 expression after hUC-MSC treatment (*n* = 4/group; ^∗^*P* < 0.05, ^∗∗^*P* < 0.01, and ^∗∗∗^*P* < 0.001 compared with one-way ANOVA with Turkey's post hoc tests). Scale bar: 50 *μ*m.

We examined whether hUC-MSCs could modulate TLR4 pathway-related agents and proinflammatory cytokine levels in rMC-1 cells. rMC-1 cells stimulated with LPS demonstrated increased GFAP (*P* = 0.0174) and TLR4 (*P* < 0.0001) expression compared with the control cells, and TLR4 was colocalized in GFAP. However, the coculture with hUC-MSCs reduced GFAP (*P* = 0.0149) and TLR4 (*P* < 0.0001) expression (Figures 7(a)–7(b)). Western blot analysis further demonstrated that hUC-MSC treatment significantly decreased TLR4 (*P* = 0.0252) expression in LPS-stimulated rMC-1 cells compared with LPS treatment alone (Figures 7(c)–7(d)). In addition, hUC-MSC treatment reduced Myd88 (*P* = 0.0134) and IL-6 (*P* = 0.0163) expression (Figures 7(c)–7(f)).

### 3.7. hUC-MSCs Modulate TLR4 Pathway-Related Agents and Proinflammatory Mediator Levels in LPS-Treated BV2 Cells

**Figure 8:**
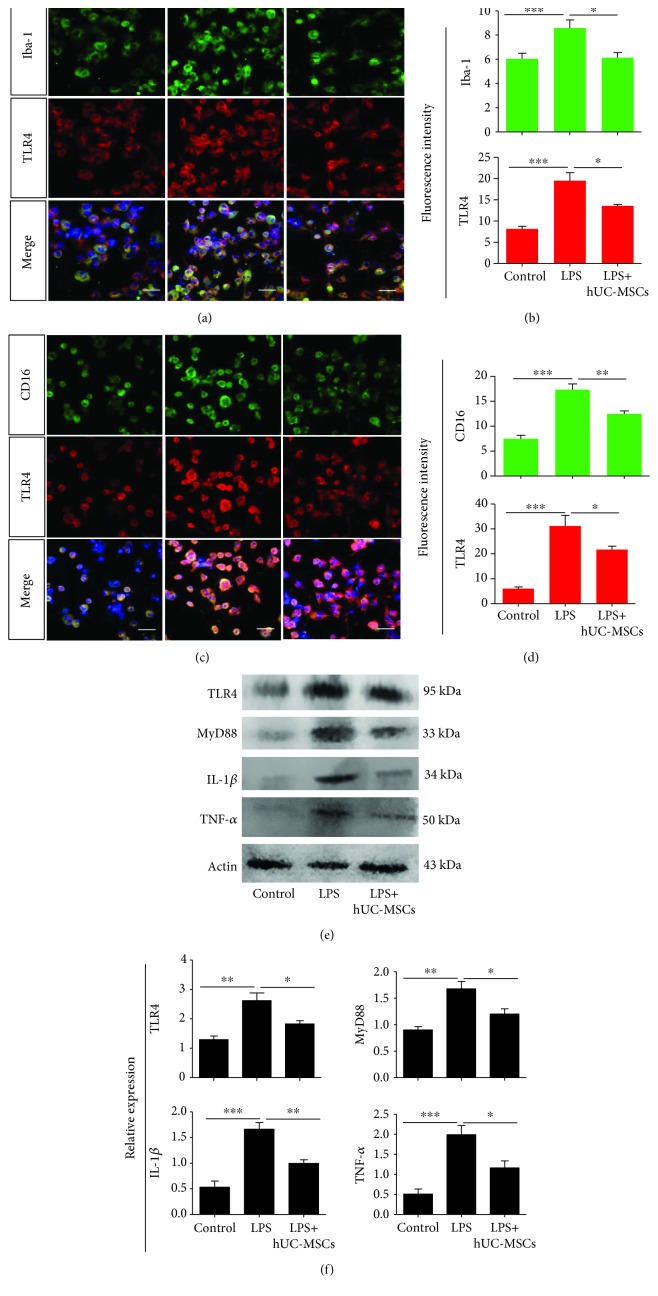
hUC-MSCs modulate TLR4 pathway-related agents and proinflammatory mediator levels in LPS-treated BV2 cells. (a and b) Double staining for iba-1 (green) and TLR4 (red), showing poor colocalization of iba1 and TLR4. The fluorescence intensity showing reduced expression of iba-1 and TLR4 in LPS-treated BV2 cells after hUC-MSC treatment (*n* = 4/group; ^∗^*P* < 0.05 and ^∗∗∗^*P* < 0.001 compared with one-way ANOVA with Turkey's post hoc tests). (c and d) Double staining for CD16 (green) and TLR4 (red) showing colocalization of CD16 and TLR4. The fluorescence intensity showing reduced expression of CD16 and TLR4 in LPS-treated BV2 cells after hUC-MSC treatment (*n* = 4/group; ^∗^*P* < 0.05, ^∗∗^*P* < 0.01, and ^∗∗∗^*P* < 0.001 compared with one-way ANOVA with Turkey's post hoc tests). (e and f) Quantification of TLR4, MyD88, IL-1*β*, and TNF-*α* expression by western blot analysis showing a significant decrease in TLR4, MyD88, IL-1*β*, and TNF-*α* expression after hUC-MSC treatment (*n* = 4/group; ^∗^*P* < 0.05, ^∗∗^*P* < 0.01, and ^∗∗∗^*P* < 0.001 compared with one-way ANOVA with Turkey's post hoc tests). Scale bar: 50 *μ*m.

We examined whether hUC-MSCs could modulate TLR4 pathway-related agents and proinflammatory cytokine levels in BV2. BV2 cells stimulated with LPS demonstrated increased iba-1 (*P* < 0.0001) and TLR4 (*P* < 0.0001) expression compared with the control cells, but with poor colocalization, whereas coculture with hUC-MSCs reduced iba-1 (*P* = 0.0191) and TLR4 (*P* = 0.0140) expression (Figures 8(a)–8(b)). In addition, our results revealed increased CD16 and TLR4 expression with obvious colocalization compared with the control cells. Coculture with hUC-MSCs reduced CD16 (*P* = 0.0080) and TLR4 (*P* = 0.0464) expression (Figures 8(c)–8(d)). Western blot analysis also demonstrated that hUC-MSC treatment significantly decreased TLR4 (*P* = 0.0275) expression in LPS-stimulated BV2 cells compared with LPS treatment alone (Figures 8(e)–8(f)). In addition, hUC-MSC treatment reduced Myd88 (*P* = 0.0264), IL-1*β* (*P* = 0.0056), and TNF-*α* (*P* = 0.0259) expression.

## 4. Discussion

**Figure 9:**
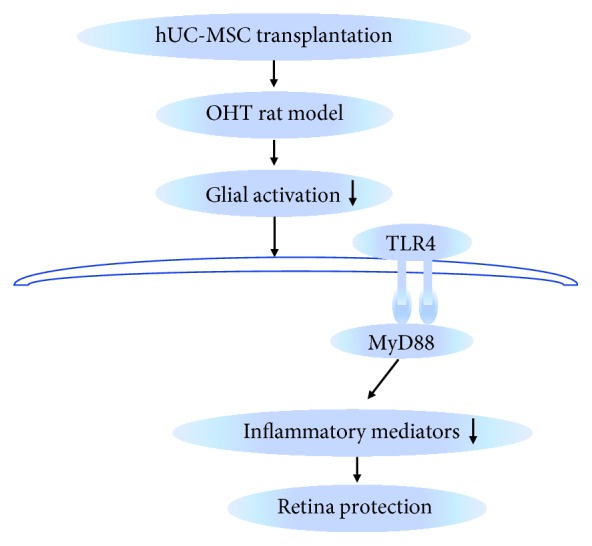
Schematic summary of the potential mechanisms for the neuroprotection of hUC-MSC transplantation in OHT rats. hUC-MSC transplantation attenuates the activation of glial cells and the TLR4-related neuroinflammatory signaling pathway in OHT rats, thereby leading to the retinal protection.

Glaucoma is an age-related multifactorial neurodegenerative disease, with an irreversible decrease in RGC injuries [35]. Many recent studies, especially from various animal models, indicate that neuroinflammation plays a role in the process of glaucoma [5, 36]. Accumulating evidence indicates that MSC transplantation is thought to be a promising treatment for RGC injury [37–39]. In addition, our previous study has shown that hUC-MSC transplantation plays a neuroprotective role in microbead-induced OHT rat models [30]. However, the exact mechanisms by which neuroinflammation is involved in hUC-MSC-treated glaucoma are poorly understood. Therefore, in our present study we investigated the possible neuroinflammation mechanisms. We investigated the changes of Müller cells, microglia, and TLR4 signaling pathways in hUC-MSC-treated OHT rat eyes. Zheng et al. have shown that the expression levels of TLR4 peaked at approximately 2 weeks and remained high for 3 weeks in optic nerve crush mice [40]. In our present study, the chronic OHT model is a relatively long-term and sustained injury model, and our previous study has demonstrated that the elevated IOP levels were sustained for at least 3 weeks after intracameral microbead injection. Therefore, we investigated the pathology and TLR4 pathway at Day 14. Our findings showed that hUC-MSC transplantation reduces the activation and proliferation of glial cells resulting from the decreased expression of GFAP and iba-1. The results were associated with the decreased expression of TLR4-signaling-involved molecules and proinflammatory cytokines in retinas. As measured by the inner retinal thickness, hUC-MSC-induced changes in retinal neuroinflammation were associated with an improvement in retina morphology compared with PBS-treated OHT eyes. Considered together, these results indicate that the amelioration of retinal neuroinflammation by hUC-MSC transplantation may be relevant to attenuating neuroinflammatory response via modulating glial cells and TLR4 signaling pathways, possibly indicating a novel repair mechanism in hUC-MSCs (Figure 9).

Similar to neurodegenerative brain diseases, OHT also leads to the neuroinflammatory response in the retina [7, 41]. Such neuroinflammatory response is associated with reactive astrocytes, Müller cells, microglia, and infiltrating monocytes. These cells act in a coordinated manner [42]. In our previous study, we demonstrated that hUC-MSC transplantation promotes the survival of the RGCs and preserves RGC function in the OHT model [30]. In our present study, though the microbead-induced OHT rat models were still implemented, we first verified the neuroprotective effect of hUC-MSCs again. Our findings revealed that hUC-MSC transplantation preserves the inner retinal thickness, which is similar to the findings of a previous study reporting that dental pulp stem cell transplants preserved RNFL thickness after optic nerve injury [43]. We then confirmed via TUNEL assay, RT-PCR, and western blot analysis that hUC-MSC transplantation decreases the apoptosis of retinal cells induced by OHT. These results were in concordance with other studies, in which different sources of MSCs inhibited the apoptosis in different RGC injury models [25, 39, 44]. In the OHT model used here, we demonstrated decreased GFAP and iba-1 levels measured in retinas at 14 days after hUC-MSC transplantation. A growing body of studies have reported that TLR4 has also been implicated in the pathogenesis of glaucoma [7, 14–16]. Therefore, our study investigated the role of hUC-MSCs in neuroinflammatory responses through the TLR4 pathway. In our study, two weeks after hUC-MSC transplantation, the expression of TLR4 was obviously decreased, which was in agreement with the results of GFAP, iba-1, and CD16 expression. Accordingly, the inhibition of the increased expression of glial cells and TLR4 may be one possible mechanism of hUC-MSCs against OHT-induced RGCs and retina injury. In the present study, we found that hUC-MSC-treated OHT eyes resulted in decreased expression of MyD88, which is a molecule involved in TLR4 signaling pathways. These results suggest that TLR4 signaling pathways may serve a vital function in attenuating retinal neuroinflammation. However, the detailed mechanisms by which TLR4 signaling pathways are suppressed by hUC-MSCs remain unclear, and additional studies should be conducted to determine this information. Glial cells in the reactive state are the main source of proinflammatory cytokines in the retina. Therefore, we further investigated the role of hUC-MSCs in inflammatory response. Our findings proved that IL-1*β*, IL-6, and TNF-*α* are markedly upregulated in OHT eyes. Importantly, hUC-MSC-related alleviation of glial cell activity and TLR4 are obviously associated with lower levels of the inflammatory cytokines. The results are similar to those from a study that revealed that the expression of inflammatory cytokines in the retina were decreased after treatment with mesenchymal stem cells derived from bone marrow and adipose [39].

Next, we examined the modulation effects of hUC-MSCs *in vitro.* In this study, we found that the coculture of hUC-MSCs reduces the expression of GFAP, TLR4, MyD88, and IL-6 in LPS-stimulated rMC-1 cells. Similarly, we also found that the coculture of hUC-MSCs reduces the expression of iba-1, TLR4, CD16, Myd88, IL-1*β*, and TNF-*α* in LPS-stimulated BV2 cells. Interestingly, we found that TLR4 is poorly colocalized with iba-1, even in OHT eyes or LPS-stimulated BV2 cells, whereas it is well colocalized with activated microglial marker CD16. In this regard, we proved that TLR4 expression is increased in active microglial cells, and that hUC-MSCs are capable of modulating both the TLR4 expression and microglial activation. Accordingly, our results provide evidence that hUC-MSCs play an important role in the activation of glial cells and the TLR4 pathway. It is unknown how the hUC-MSCs modulate the TLR4 pathway in our present study; however, evidence has indicated that MSC-Exo has immunomodulatory properties [20, 45, 46]. For example, Zhao et al. demonstrated that miR-182 in MSC-Exo regulated the macrophage phenotype via the TLR4/NF-*κ*B pathway [46]. Henao et al. showed that MSC-Exo regulated an internal proinflammatory program in activated macrophages in acute peritonitis [45]. Our future work will concentrate on determining the role of hUC-MSC-Exo in TLR4 pathway modulation.

Although preclinical studies provide encouraging results that suggest that MSCs are promising therapies for glaucoma [30, 38, 39, 43, 44], safety remains one of the main concerns in cell therapy. Recent reports of the adverse effects of “stem cells,” such as ocular hypertension, retinal atrophy, and the induction of retinal detachments via proliferative vitreoretinopathy leading to severe visual loss, have raised some concerns regarding the existing safety of stem cell transplantation [47, 48]. Of note, these activities did not require the new drug application investigation by the U.S. Food and Drug Administration (FDA) [49]. Therefore, to eventually generate safe and efficacious stem-cell-based therapeutic approaches, the production of safe cells, and corresponding quality control systems, delivery techniques, and the strict following of the guidelines for the Clinical Translation of Stem Cells must be applied for assuring the safety and efficiency of the stem cells.

## 5. Conclusion

Our previous study has already shown that hUC-MSCs reveal a neuroprotective role by the secretion of neurotrophic factors rather than cell replacement in OHT rats [30]. Here, hUC-MSC transplantation inhibits the activation and expression of glial cells and TLR4-related neuroinflammatory pathways to attenuate retinal neuroinflammation in OHT rats, proving again that the neuroprotection of hUC-MSCs in OHT rats acts in a bystander-like way. In summation, our present study provides novel evidence that hUC-MSC transplantation attenuates OHT-induced retinal neuroinflammation via the TLR4 pathway.

## Figures and Tables

**Table 1:** List of PCR primers and the primary antibodies.

Gene	Oligonucleotides	Primary antibodies
*Bax*	F: AGTGTCTCAGGCGAATTGGC R: CACGGAAGAAGACCTCTCGG	Ab 32503 (Abcam, UK)

*TLR4*	F: CTCACAACTTCAGTGGCTGGATTT R: TGTCTCCACAGCCACCAGATTC	Ab 13556 (Abcam, UK)

*MyD88*	F: AGGAGATGGGTTGTTTCGAGTAC R: CTCACGGGTCTAACAAGGCTA	NB 100-56698 (Novus International, USA)

*IL-1β*	F: TGCTGTCTGACCCATGTGAG R: GGGGAACTGTGCAGACTCAA	NB 600-633 (Novus International, USA)

*IL-6*	F: CCTTCGGTCCAGTTGCCTT R: AGAGGTGAGTGGCTGTCTGT	NB 600-113 (Novus International, USA)

*TNF-α*	F: ATGACGGTGGTGAGCTTTCA R: TAACCCGTCCGTGAGACTTG	NB P1-19532 (Novus International, USA)

*Actin*	F: CCCATCTATGAGGGTTACGC R: TTTAATGTCACGCACGATTTC	4970s (CST, USA)

## Data Availability

The data used to support the findings of this study are available from the corresponding author upon request.

## Supplementary Materials

Supplementary Materials Figure S1: microsphere-induced OHT rats had markedly higher IOP levels. IOP changes in 2 weeks in the study groups. Data represent mean ± SEM from *n* = 10 rats. ^∗^*P* < 0.05 compared to the normal control group.
