# Supplementary material for: Human Umbilical Cord Mesenchymal Stem Cells Attenuate Ocular Hypertension-Induced Retinal Neuroinflammation via Toll-Like Receptor 4 Pathway
Source: Stem Cells Int. 2019 Oct 15;2019:9274585. doi: 10.1155/2019/9274585 (PMC6815608; doi:10.1155/2019/9274585)
Supplement: Supplementary Materials — Figure S1: microsphere-induced OHT rats had markedly higher IOP levels. IOP changes in 2 weeks in the study groups. Data represent mean ± SEM from n = 10 rats. ∗P < 0.05 compared to the normal control group. [file 9274585.f1.pdf]

Supplementary Materials

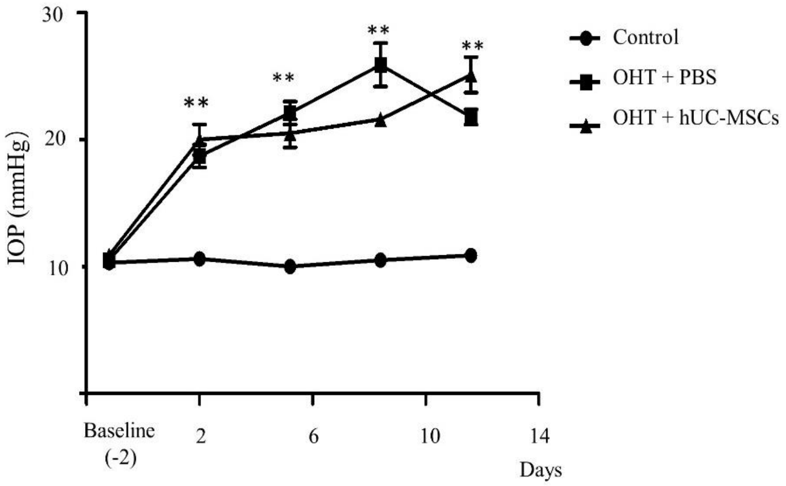

Figure S1. Microspheres induced OHT rats had markedly higher IOP levels. IOP changes in 2 weeks in the study groups. Data represent mean ± SEM from n=10 rats. $*p < 0.05$ compared to normal control group.
